# Regulation of HLA class I expression by non-coding gene variations

**DOI:** 10.1371/journal.pgen.1010212

**Published:** 2022-06-06

**Authors:** Florence Bettens, Halit Ongen, Guillaume Rey, Stéphane Buhler, Zuleika Calderin Sollet, Emmanouil Dermitzakis, Jean Villard

**Affiliations:** 1 Transplantation Immunology Unit and National Reference Laboratory for Histocompatibility, Geneva University Hospitals, Geneva, Switzerland; 2 Department of Genetic Medicine and Development, University of Geneva, Geneva, Switzerland; Finnish Red Cross Blood Service, FINLAND

## Abstract

The Human Leukocyte Antigen (HLA) is a critical genetic system for different outcomes after solid organ and hematopoietic cell transplantation. Its polymorphism is usually determined by molecular technologies at the DNA level. A potential role of HLA allelic expression remains under investigation in the context of the allogenic immune response between donors and recipients. In this study, we quantified the allelic expression of all three HLA class I loci (HLA-A, B and C) by RNA sequencing and conducted an analysis of expression quantitative traits loci (eQTL) to investigate whether HLA expression regulation could be associated with non-coding gene variations. HLA-B alleles exhibited the highest expression levels followed by HLA-C and HLA-A alleles. The max fold expression variation was observed for HLA-C alleles. The expression of HLA class I loci of distinct individuals demonstrated a coordinated and paired expression of both alleles of the same locus. Expression of conserved HLA-A~B~C haplotypes differed in distinct PBMC’s suggesting an individual regulated expression of both HLA class I alleles and haplotypes. Cytokines TNFα /IFNβ, which induced a very similar upregulation of HLA class I RNA and cell surface expression across alleles did not modify the individually coordinated expression at the three HLA class I loci. By identifying *cis* eQTLs for the HLA class I genes, we show that the non-coding eQTLs explain 29%, 13%, and 31% of the respective HLA-A, B, C expression variance in unstimulated cells, and 9%, 23%, and 50% of the variance in cytokine-stimulated cells. The eQTLs have significantly higher effect sizes in stimulated cells compared to unstimulated cells for HLA-B and HLA-C genes expression. Our data also suggest that the identified eQTLs are independent from the coding variation which defines HLA alleles and thus may be influential on intra-allele expression variability although they might not represent the causal eQTLs.

## Author summary

In transplantation, the allogenic immune response is linked to the HLA compatibility between donor and recipient, HLA genes being highly polymorphic. The impact of this allelic polymorphism on MHC cell surface expression and the potential role of expression in the T cell activation and on clinical outcomes remains poorly investigated. In this study, we documented the individual variability of allelic HLA class I expression in PBMCs by RNA sequencing. To mimic the inflammatory clinical situation after transplantation, we performed a similar analysis following cytokine (TNFα/IFNβ) stimulation of PBMCs. The results demonstrated a coordinated and paired expression of both alleles of the same locus in all individuals. The levels of expression of matched HLA-A~B~C haplotypes differed in distinct PBMCs suggesting that expression of both HLA class I alleles and haplotypes is regulated individually. To identify *cis* regulatory elements of expression we performed an eQTL analysis on unstimulated and stimulated PBMCs. These eQTLs accounted for up to 9%, 23% and 50% of the respective HLA-A, B and C expression variance in stimulated PBMCs. Of interest, we could show that they are independent from the SNPs encoding allelic variation, meaning that they could explain a different portion of the variance in HLA expression.

## Introduction

HLA class I molecules are expressed constitutively on nucleated cells and function as antigen presenting molecules to cytotoxic T cells in immune responses to pathogens, cancer cells and autoantigens [1]. They also regulate the innate immune response by influencing NK cell activation [2]. HLA disparities between donors and patients induce allogenic immune responses, leading to rejection or graft versus host disease (GVHD) in different transplantation settings [3]. HLA class I molecules are characterized by an extremely high polymorphism and variable levels of expression [4–7], which potentially influence their function(s). For instance, tumor cells downregulate HLA expression to escape immune-surveillance by T cells [8,9]. *In vitro* allogenic immune responses were previously shown by us and others [10–12] to be partially dependent on HLA-C expression. Intra- and inter-individual variation of HLA cell surface expression on T and B cells was also described to impact antibody dependent cytotoxic immune response [13]. More recently, HLA-expression was also shown to impact crossmatch outcomes in transplantation diagnostic [14]. Two retrospective clinical studies tested the impact of HLA expression on clinical outcome in the setting of HLA-C mismatched unrelated hematopoietic stem cell transplantation (HSCT). Both studies used as proxy of HLA-C expression the mean cell surface expression reported by Apps et al. [4]. While Petersdorf et al [15] found an association between highly expressed HLA-C*03 or C*14 allotypes and increased mortality, Morishima et al [16] did not. In other clinical settings, a high HLA-C expression was correlated to more efficient recognition of HIV by cytotoxic cells and lower viremia in patients [4,17], while lower levels of HLA-C expression had a protective effect from Crohn’s disease [7]. Similarly, higher HLA-B*27 expression levels were reported in patients with ankylosing spondylitis compared to healthy donors [18].

HLA expression regulation underlies complex mechanisms, involving genetic polymorphisms as well as transcriptional and translational factors [19–23]. To date, no consensus explanation exists for the variable levels of HLA expression that are observed [24].

Initial reports on HLA class I expression were based on cell surface and gene expression and relied on allotypes, without the possibility to discriminate between the two alleles carried by heterozygous donors at a given locus. [4,7,17,25,26]. The advent of more recent technologies using RNA sequencing has allowed qualitative, quantitative and equivalent inter-allelic expression analyses [14,27–30].

An extended analysis of single nucleotide polymorphisms (SNPs) in the non-coding regions of HLA genes, which tag transcript abundance (expression quantitative traits loci, eQTLs) was performed by Aguiar et al. in lymphoblastic cells of the GEUVADIS consortium [28]. They identified HLA-C associated eQTL’s in strong linkage disequilibrium with the previously described genetic variants rs9264942 and rs2395471 and deletion (263del/ins) [17,31,32]. Vandiedonck et al. 2011 [33] analyzed the specific transcriptional variation of haplotypes associated with autoimmune diseases in three known homozygous cell lines PGF, COX and QBL.

In the present study, HLA class I allelic RNA expression, analyzed in peripheral blood mononuclear cells (PBMCs) of healthy blood donors, was quantified using RNA sequencing. To mimic clinical situations with inflammation driven by events such as alloreactivity, auto-immunity or infection, the analyses were performed on PBMCs before and after stimulation with the pro-inflammatory cytokines Tumor Necrosis Factor alpha (TNFα) and Interferon beta (IFNβ). Furthermore, we investigated whether variations in the non-coding genome (eQTLs) affect HLA class I expression. The eQTLs were first analyzed on T cells (169 samples) from the Blueprint consortium and then compared to the ones retrieved in PBMCs of the current study (54 samples), before and after stimulation.

## Results

### HLA class I expression at the allelic and individual levels

**Fig 1:**
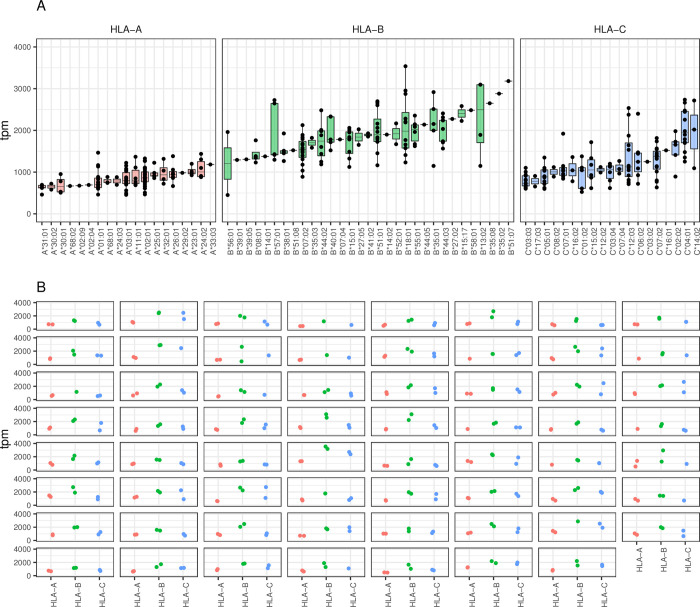
Gradient HLA class I expression at the allelic level. Panel (A) HLA class I RNA expression measured in samples of PBMCs obtained from 63 individuals is plotted as tpm (transcript per million) per allele (indicated on the horizontal axis) for HLA-A, B and C. Nine-teen HLA-A, 31 HLA-B and 19 HLA-C unique alleles are detected. Mean expressions are 866±224, 1870±541and 1238±532 tpm for HLA-A, B and C, respectively. Each dot represents the expression of an allele in one individual. Note that the HLA typing inferred from RNA sequencing corresponded to the available high-resolution typing performed on DNA. Panel (B) Allelic expression is plotted per locus (horizontal axis) and per individual. Each facet represents one individual and each dot one allele.

To evaluate and compare the expression of HLA class I genes, RNA from 63 healthy blood donors was isolated from PBMCs and sequenced. Expression levels for the alleles detected in our cohort are provided in Fig 1A. The highest mean expression was measured for HLA-B alleles (1870±541 transcripts per million, tpm) followed by HLA-C (1238±532 tpm) and HLA-A (866±224 tpm) alleles. Variation in expression was also the highest among HLA-B alleles with a maximum fold variation ratio of 7.86 between HLA-B*18:01 and B*56:01 which comprise the highest and lowest tpm values, respectively. The expression of HLA-A alleles was more even with a ratio of 3.2 between the highest (A*01:01) and lowest (A*31:01) tpm values. HLA-C was intermediate with a ratio of 5.2 between the highest (C*04:01) and lowest (C*01:02) tpm values. A more conservative comparison of expression between alleles using median values showed the highest fold ratio for HLA-C (2.7) between C*14:02 and C*03:03, followed by HLA-B (2.0) between B*13:02 and B*56:01 and HLA-A (1.8) between A* 33:03 and A*31:01. Expression according to HLA antigens is shown in S1 Fig.

**Fig 2:**
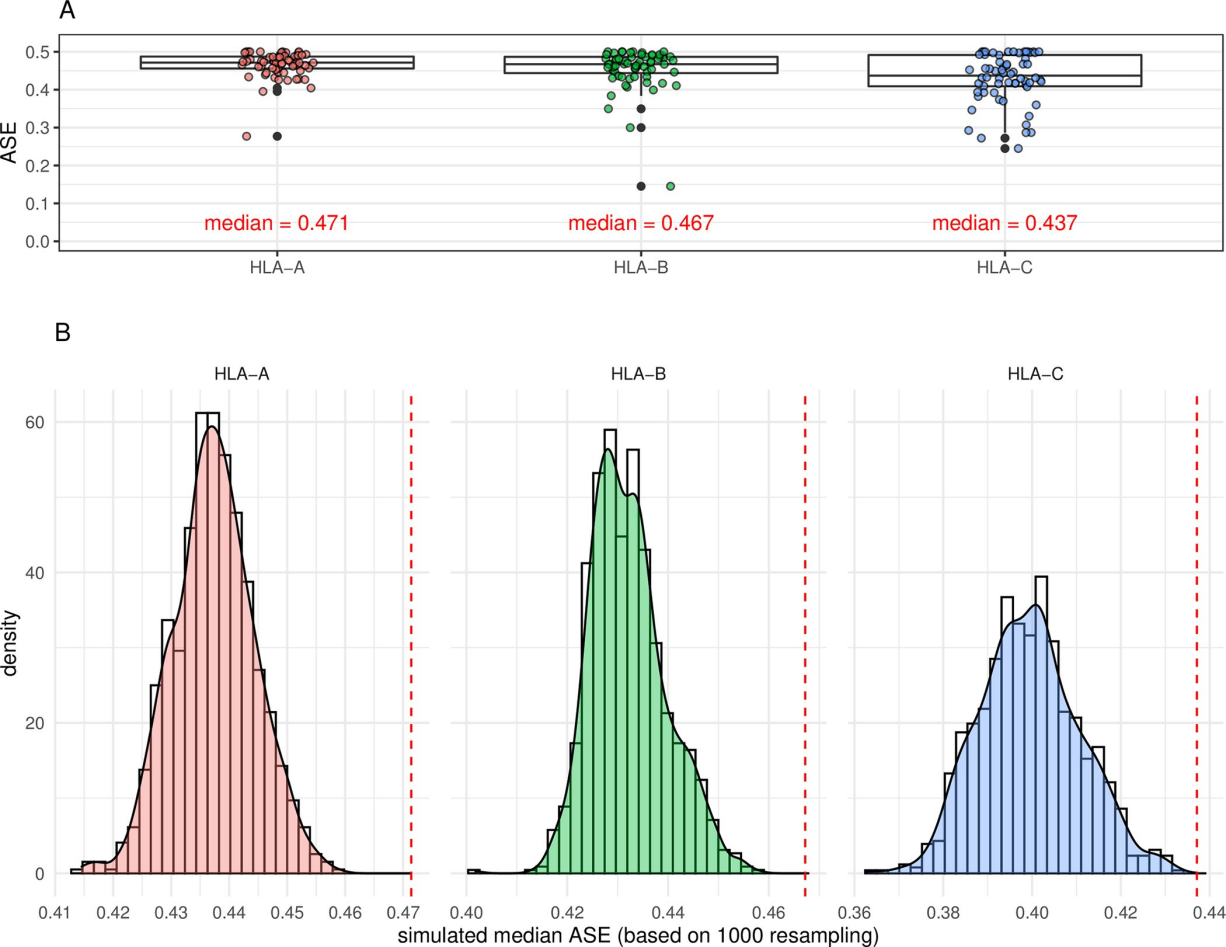
ASE and permutations. Panel (A) Allele specific expression (ASE) of the lowest expressed allele in relation to the expression of both alleles for the three HLA class I genes is plotted. Each dot represents one individual. The observed median is also shown for each locus. Panel (B) Empirical distributions of simulated ASE following a permutation procedure. In this approach, tpm values were shifted randomly among individuals if belonging to the same allele (i.e., permuting the observed tpm values among all carriers of a given allele and doing so for the whole cohort simultaneously). The procedure was replicated 1000 times and for each replicate ASE was computed in every individual for the three loci. The simulated median ASE was computed at each locus and plotted along a density curve and according to a discrete histogram distribution. The dotted red line represents the observed median ASE at a given locus (see panel (A) in comparison to the empirical distribution obtained through the resampling process.

HLA expression per locus and per donor is represented in Fig 1B. A coordinated expression of both alleles is clearly visible in each heterozygous donor for the three HLA class I genes. The intra-individual variation of expression was the lowest at HLA-A and the highest at HLA-C. The intra-individual variation at HLA-B was also low except in two donors. Along this line, a high correlation between pairs of alleles in heterozygotes at HLA-A, and B and a slightly lower correlations at HLA-C was measured (HLA-A: spearman ρ = 0.66 p = 3.6x10^-7^, HLA-B: ρ = 0.67 p = 1.0x10^-9^ and HLA-C: ρ = 0.51 p = 1.7x10^-5^) (S2 Fig). Calculation of allele specific expression (ASE) as the ratio of the lowest expressed allele towards the total expression of both alleles at a given locus in each individual revealed a very balanced pattern of expression with median values of 0.47, 0.47 and 0.44 for HLA-A, B and C, respectively (Fig 2A). For one individual, the ASE was exceptionally low (i.e., 0.1) due to the low expression of one HLA-B*56:01 allele. Furthermore, in order to test whether the balanced pattern of expression of HLA class I alleles was different from what could be expected by chance, we applied a resampling procedure. The empirical distributions of the simulated ASE obtained after 1000 permutation replicates are shown in Fig 2B (see the figure legend for more details). The observed ASE at HLA-A, B and C are significantly closer to the maximum possible balance of expression (i.e., 0.5) than any of the replicates (p-values < 0.001).

### Upregulated HLA expression

**Fig 3:**
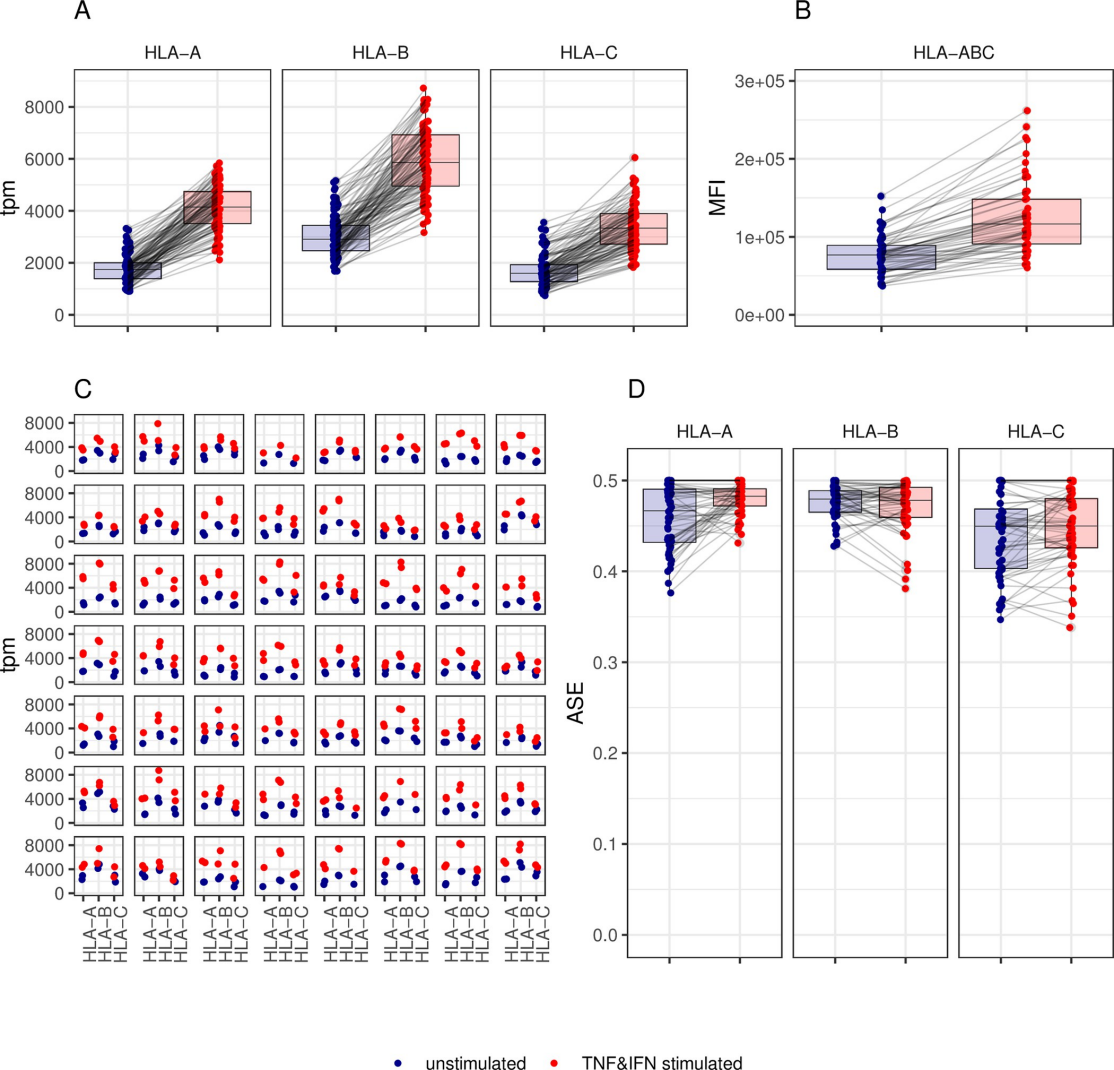
Upregulation of expression induced by TNFα and IFNβ. Panel (A) RNA expression of HLA class I alleles (given as tpm) is plotted in 56 PBMC samples stimulated overnight with (red dots) or without (blue dots) pro-inflammatory cytokines TNFα and IFNβ Each dot represents one allele of one PBMC sample. Panel (B) Cell surface expression of HLA-ABC as determined by flow cytometry is plotted and expressed as MFI for the same PBMCs tested just prior to RNA extraction. Panel (C) Allelic expression is plotted per locus (horizontal axis) and per individual. Each facet represents one individual, each dot one allele. The dots are colored according to the same code as in Panel (A). Panel (D) Allele specific expression (ASE) at the three HLA class I genes is plotted according to both stimulation conditions.

To further investigate the influence of pro-inflammatory cytokines on HLA expression, PBMCs of 56 blood donors were stimulated overnight without or with the cytokines TNFα and IFNβ. Their respective expression was determined and is shown in Fig 3. Mean expression of HLA-A, B and C alleles was upregulated to a similar extent (Fig 3A). Mean fold upregulation was 2.5, 2.04 and 2.11 for the tested HLA-A, B and C alleles, respectively, with spearman ϱ coefficient of 0.4, 0.31 and 0.58 and p-values of 2.9x10^-4^, 1.0x10^-3^ and 2.2x10^-16^. The detailed analysis of the upregulation per allele is shown in S3 Fig and revealed only a few possible outliers. Namely, HLA-A*29:02, 30:01, 32:01 and HLA-C*16:01 had higher fold expression of 4.1, 3.4, 3.5 and 2.7, respectively, while HLA-B* 15:01 had a lower fold expression of 1.3 following stimulation. Otherwise, although the results were gathered from 56 different PBMC cultures performed at different times, a very homogenous and conserved upregulation of expression was observed among alleles. In accordance with RNA expression, cell surface expression of the corresponding HLA-class I molecules was upregulated to a similar extent with a ratio of mean fold upregulation of 1.6, as determined by flow cytometry (Figs 3B and S4). The Spearman correlation for HLA cell surface expression between cells stimulated or non-stimulated with cytokines was 0.84 with a p-value of 1.0x10^-16.^ The coordinated expression of alleles in heterozygous donors persisted after induced upregulation (Figs 3C and S5). Moreover, the balance of expression among alleles within individuals, as measured by ASE, was similar to the data previously shown in Fig 2 for the uncultured PBMCs, with median ASE comprised between 0.45 and 0.48 in unstimulated and stimulated PBMCs (Fig 3D). Upregulation through overnight cytokines stimulation did not change the much conserved balance of expression for HLA-B and C or even slightly reinforced it as observed for HLA-A (paired Wilcoxon rank sum test, p-value < 0.001, median ASE for unstimulated PBMCs = 0.467, median ASE for stimulated PBMCs = 0.483).

### Allelic expression in HLA-A~B~C~DRB1 haplotypes

**Fig 4:**
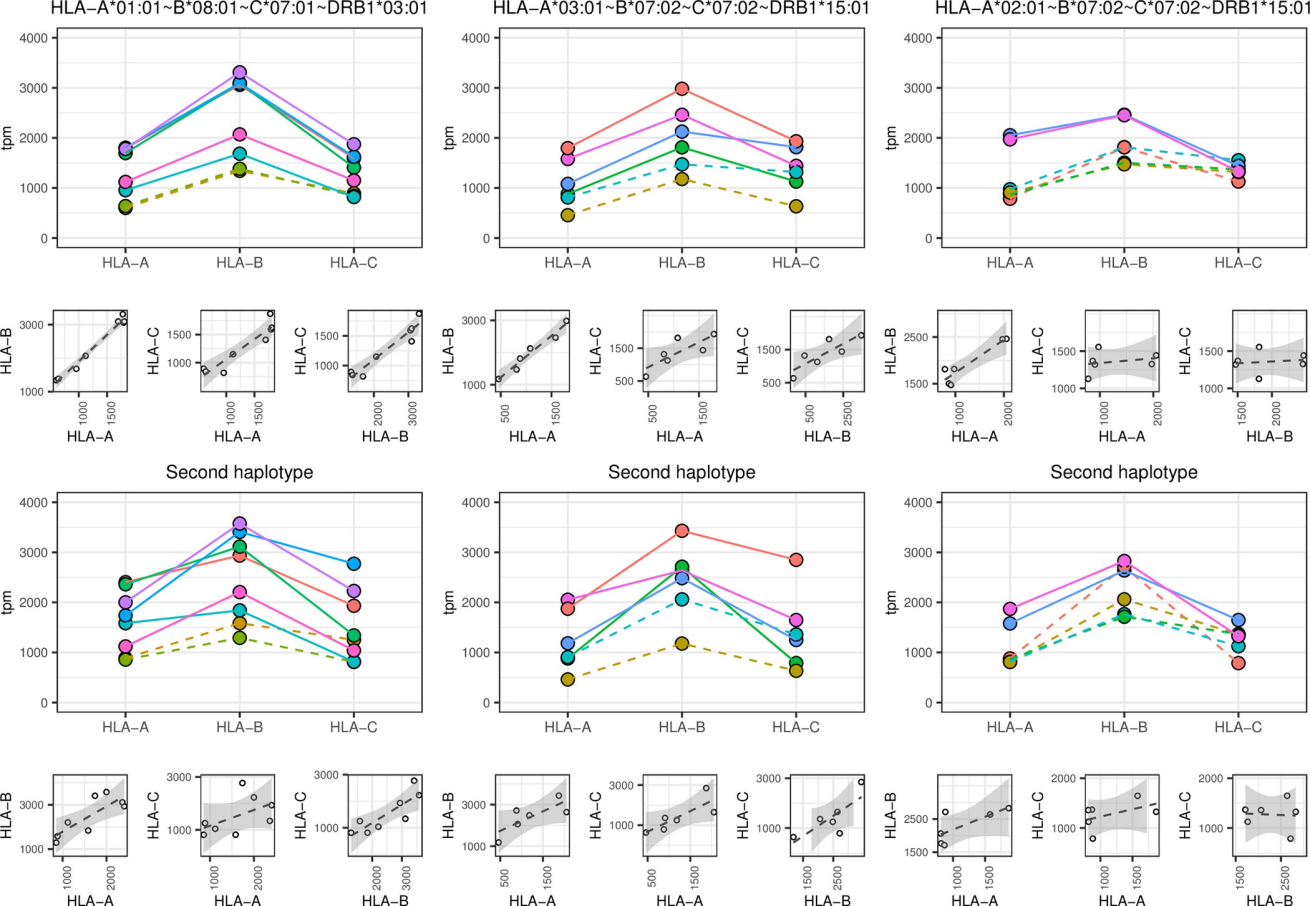
Allelic expression in PBMC carrying common HLA haplotypes. The upper panels represent the RNA expression (in tpm) of alleles segregating on three common HLA haplotypes as indicated. In the lower panels, the RNA expression (in tpm) of the alleles belonging to the second haplotype is shown. Each individual carrying a given haplotype is shown with a different color code. Straight and dotted lines correspond to RNAs of cells kept in culture without stimulation overnight or of freshly isolated PBMCs, respectively. The HLA-typing of the second haplotype differ between samples sharing a common haplotype. Small facets represent the pairwise linear regression of expression between loci. The standard error of the regression is indicated as grey shedding.

The RNA expression measured at the allelic level in PBMCs was grouped according to the expected segregation of alleles on three common HLA haplotypes (i.e. inferred at least five times in our cohort and estimated with a frequency above 1% in a large Swiss cohort [34]. The three common haplotypes considered were HLA-A*01:01⁓B*08:01⁓C*07:01⁓DRB1*03:01, HLA-A*03:01⁓B*07:02⁓C*07:02⁓DRB1*15:01 and HLA-A*02:01⁓B*07:02⁓C*07:02⁓DRB1*15:01. The RNA expression of alleles within the common haplotypes in freshly isolated PBMCs or in cells kept in culture without stimulation overnight are shown in the upper panels of Fig 4. A stacked expression was seen across loci for alleles belonging to the same haplotype. In contrast, the corresponding expression of alleles on the second carried haplotype was less concordant as these haplotypes differed among individuals sharing the same common haplotype (Fig 4 lower panels). This is also suggested by the broader standard errors of regression between pairs of loci for the second carried haplotype when compared to the common one (Fig 4, small facets).

### Expression quantitative loci analysis of the HLA class I region

Since the PBMC dataset generated in this study has a small sample size, eQTL analyses were carried out also on an external, larger, dataset comprising T cells from the Blueprint consortium (www.blueprint-epigenome.eu).

Genome-wide significant eQTLs (FDR = 5%, Table 1) were identified in 294, 428, and 7502 genes in unstimulated PBMCs, stimulated PBMCs, and Blueprint T cells, respectively.

**Table 1:** *Cis* eQTL.

Cell Type	No of Samples	No of Genes	eQTLs FDR 5%
PBMC unstimulated	55	19117	294
PBMC stimulated	55	18737	428
Blueprint T Cells	169	15488	7202

**Fig 5:**
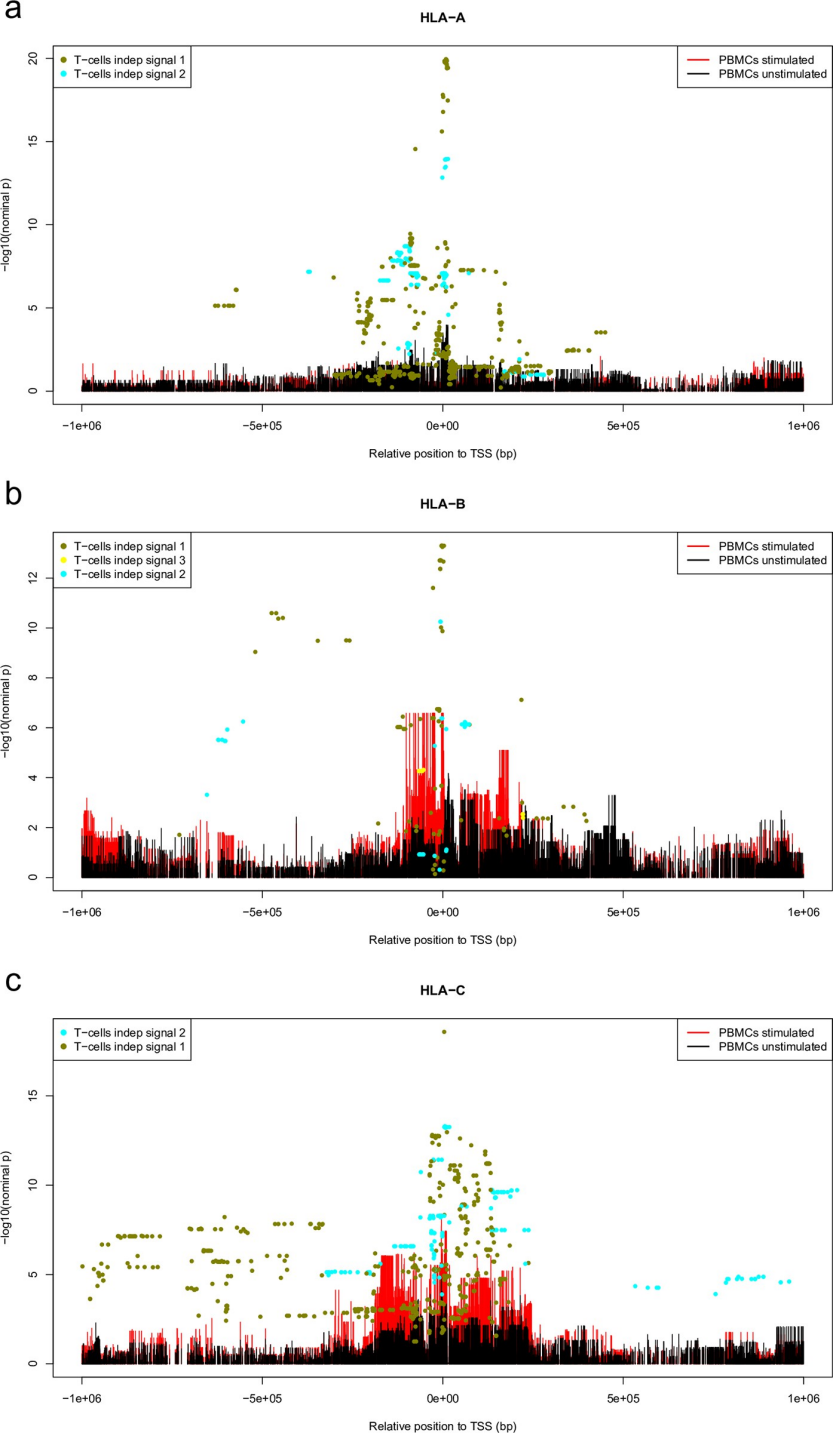
The genomic locations of the eQTLs. The genomic locations of the eQTL associations in the *cis* regions (± 1 Mb from the transcription start site) of HLA-A (**a**), HLA-B (**b**), and HLA-C (**c**). The x-axes are the relative position of the variants to the TSS, and the y-axes represent the significance of the eQTL association (-log10 nominal p-value). The colored bars show all the variants in the region and their eQTL p-values in stimulated and unstimulated PBMC cells. The colored points represent all the variants for each of the genome-wide significant independent eQTL signals in Blueprint T cells.

**Fig 6:**
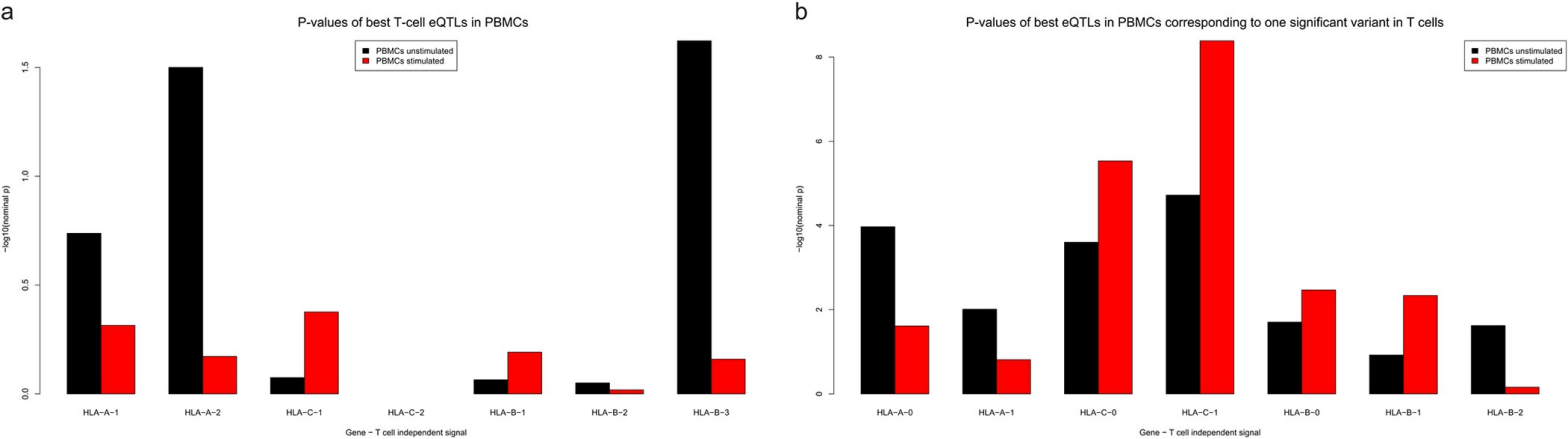
Comparison of best SNPs between T cells, unstimulated PBMCs and stimulated PBMCs. (**a**) The -log10 nominal p-values of the best variant for a T-cell independent signal in PBMCs. (**b**) The -log10 nominal p-values of the best PBMC variant amongst all the significant variants for each of T-cell independent signals.

There are no genome-wide significant eQTLs for any of the HLA class I genes in unstimulated PBMCs. Stimulated PBMCs have a genome-wide significant eQTL for HLA-B (6:31370329:C:A, p = 2.6e-7) and HLA-C (6:31243785:G:T, p = 4.1e-9) genes. In Blueprint T cells, 2, 3, and 2 independent eQTLs for HLA-A, B, and C, were identified respectively (Table 2 and Fig 5). The nominal p-values of the T-cell eQTLs in PBMCs for the best eQTL variant per independent signal are shown in Fig 6A. The p-values of the best eQTL variant in PBMCs that correspond to one of the significant variants in T cell independent signals are shown in Fig 6B. Two SNPs seen as significant in T cells were also seen in unstimulated PBMCs as significant (Fig 6A) and the best SNP’s seen in unstimulated and stimulated PBMCs are also seen in T cells (Fig 6B). We tested how well all T-cell eQTLs are replicated in the PBMCs using the π_1_ estimates ([35]) and estimated that of the T-cell eQTLs, 34% are active in unstimulated PBMCs, and 38% are active in stimulated PBMCs. To perform the aforementioned π_1_ statistic the best eQTL found in stimulated PBMC which corresponds to one of the most significant eQTLs of the Blueprint T-cell were chosen.

**Table 2:** Best eQTL SNPs shared between T cells and PBMCs.

Gene	HLA-A		HLA-B			HLA-C	
**Independent Signal**	HLA-A-1	HLA-A- 2	HLA-B-1	HLA-B-2	HLA-B-3	HLA-C-1	HLA-C-2
**T cell SNP**	6: 29917673:G:A	6: 29923522:C:T	6:31327723:G:A	6:31331829:C:T	6:31378510:G:A	6:31236051:G:A	6:31241002:A:G
**T cell p-value**	9.42E-14	4.31E-07	3.46E-09	8.81E-07	1.26E-05	5.53E-19	1.87E-08
**Unstimulated PBMC p-value**	0.1828	0.0316	0.8610	0.8904	0.0239	0.8416	NA
**Stimulated PBMC p-value**	0.4846	0.6724	0.6432	0.9589	0.6924	0.4203	NA
**Independent Signal**	HLA-A-0	HLA-A-1	HLA-B-0	HLA-B-1	HLA-B-2	HLA-C-0	HLA-C-1
**Best SNP in unstimulated PBMCs** ^**1)**^	6:29920713:T:C	6:29918841:G:A	6:31328795:A:T	6:31347798:G:A	6:31381533:C:T	6:31240712:A:C	6:31243785:G:T
**Best SNP unstimulated PBMCs p-value**	0.0001	0.0097	0.0197	0.1194	0.0239	0.0002	1.90E-05
**Best SNP in stimulated PBMCs** ^**1)**^	6:29910801:C:A	6:29892854:T:G	6:31334945:T:C	6:31347798:G:A	6:31378510:G:A	6:31274027:C:T	6:31243785:G:T
**Best SNP stimulated PBMCs p-value**	0.0243	0.1540	0.0034	0.0046	0.6924	2.93E-06	4.11E-09

^1)^ the best eQTL in unstimulated or stimulated PBMCs that correspond to T cell eQTL’s

The eQTLs identified account for 29%, 13%, and 31% of the variance in HLA-A, B, C expression in unstimulated cells, respectively, and 9%, 23%, and 50% of the variance in stimulated cells. Note that as described in Table 2, there are no significant eQTLs for the unstimulated cells and HLA-A is not significant for the stimulated cells. In order to test whether this variance is mostly independent of the variance of the coding variants that determine an individual’s HLA allele, the pairwise correlation was calculated between the eQTL variant and the coding variants for the HLA alleles found in 1000 European genomes of this study. The correlation between the eQTLs and the coding variants that dictate HLA-alleles are low (S6 Fig) indicating that the intra-HLA allele expression variance may be explained by the non-coding variants involved in the regulation of HLA class I gene expression.

**Fig 7:**
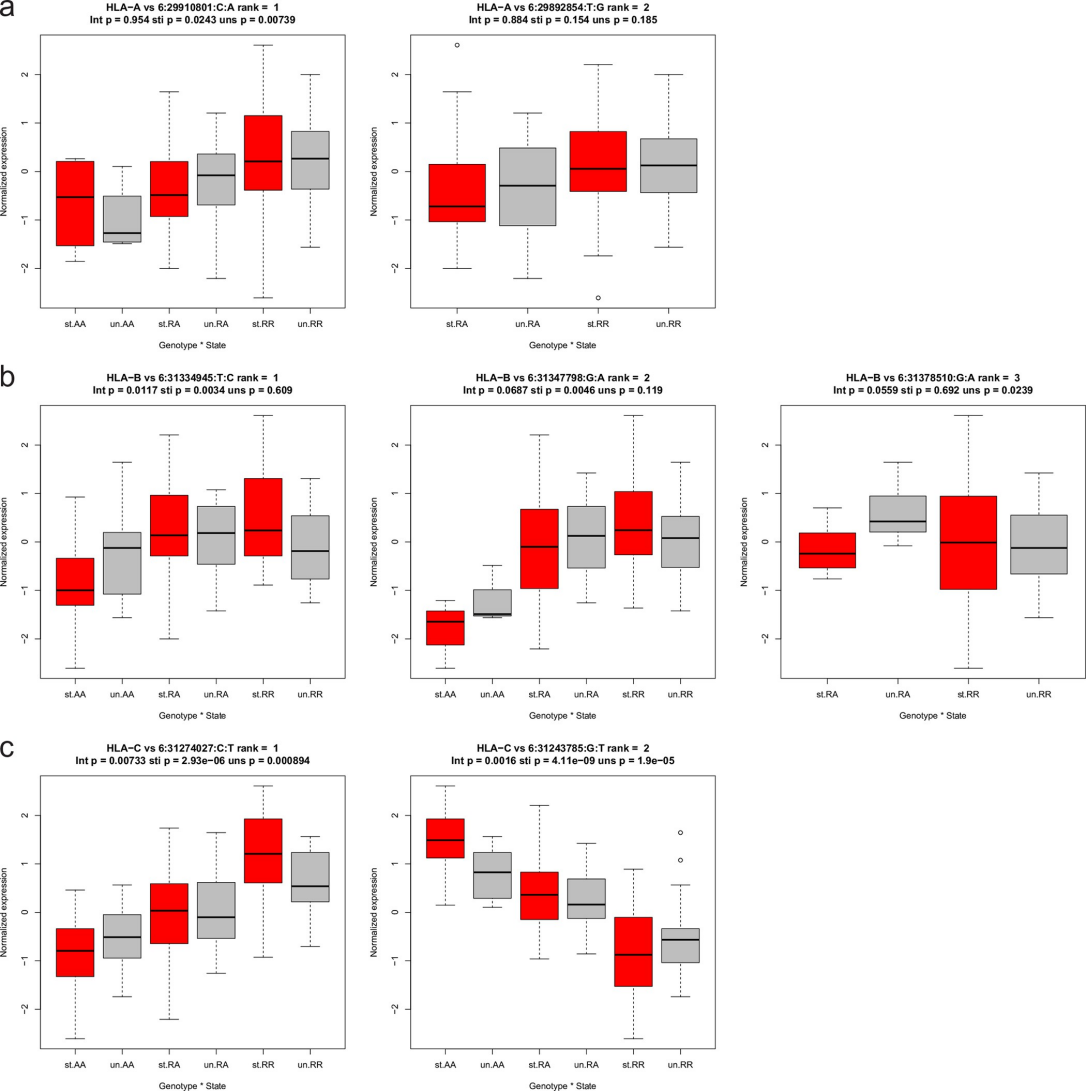
Association between eQTL genotypes and HLA expression in unstimulated versus stimulated PBMCs. Interaction analysis boxplots showing the association between the independent eQTL genotypes (AA = homozygous alternative, RA = heterozygous, RR = homozygous reference) and normalized expression for HLA-A (**a**), HLA-B (**b**), and HLA-C (**c**). The stimulated cells are shown in red, and the unstimulated cells are in grey. The interaction p-value and the p-values in stimulated and unstimulated cells are provided in the titles of the boxplots.

Lastly, significant difference in the effect sizes of HLA class I eQTLs between unstimulated and stimulated PBMCs was assessed. We used a linear mixed model to check the interaction between genotype and stimulation status of the samples. We observed that one, two and two eQTLs for HLA-A, B and C, respectively, show significantly higher effect sizes in stimulated cells vs. unstimulated cells (Fig 7). For one signal in HLA-B and all signals in HLA-C significant interaction p-values were observed, indicating that there is a significant difference between the effect sizes of these variant in unstimulated vs. stimulated cells.

## Discussion

HLA expression at the cell surface might influence the alloreactivity induced in transplantation when T cells recognize non-self HLA. Indeed, Petersdorf et al [15] found an association between highly expressed HLA-C allotypes and increased mortality, while Morishima [16] et al did not. Likewise, high expression of HLA class II (HLA-DPB1) antigens is reported to be risk factor for developing acute graft versus host disease in HSCT [36–38].

In this study, we quantified allelic expression of the three HLA class I genes on an individual basis by RNA sequencing and analyzed whether non-coding genome variations like *cis* eQTL’s could explain the observed levels of expression in PBMCs. HLA expression in T cells of the Blueprint Consortium was additionally investigated to increase sample size and the upregulated expression in cytokine-stimulated PBMCs was measured to allow a better balance of the results towards expression. We focused on HLA class I alleles only, as they are constitutively expressed in all PBMCs in contrast to HLA class II alleles, which are only constitutively expressed in specific cell types such as B cells and professional antigen presenting cells. HLA class II allele expression might thus be dependent on subpopulation distribution and cell activation status in PBMCs. Note that Yamamoto et al [29] as well as Johansson et al [30] did not consider this caveat when they analyzed HLA class II expression by RNA-Seq capture methodology in PBMC samples. *Cis* regulating elements associated to HLA-DQB1 allele-specific expression variability after autoimmune T-cell activation were reported by Gutierrrez et al [39]. In contrast to their publication, we were not able to define dynamic ASE (dynASE), which can be associated with HLA expression. We speculate that the power to detect these dynASE sites could be due, on one side, to the difference between HLA class I and HLA class II expression specificity and, on the other side, to the time course experiment they performed with 8 time points, which we do not have in our cohort.

Our results confirm the variability of expression among HLA class I alleles as previously reported [5,14,28,30,40–42]. Nevertheless, considering the mean expression of alleles at the locus level, our data are not in agreement with other publications classifying HLA-C mRNA expression as the lowest [30,43]. Furthermore, the levels of expression that we observe across distinct alleles differ from the values reported in some publications [5,30,40,42], while they concord, with the results published by Garcia-Romano et al [44] and Yamamoto et al [29], who suggest like us that HLA-B alleles possesses the highest and HLA-A alleles the lowest mean expression, respectively. Due to the limited numbers of samples analyzed, the study was not designed to evaluate the specific expression of alleles at each locus. Lee et al [42] reported expression of allele with more than 10 individuals per allele in the GEUVADIS cohort. Nevertheless, if we classify the alleles as high or low expressers, we obtain similar subgroups to the ones reported by others [4,29,42]. For example, the highest/lowest mean expression among HLA-C alleles was seen for HLA-C*04 and 14 and C*03 (Fig 1), respectively. While the lowest HLA-B expression was seen for the HLA-B*56 allele. This steadiness of expression at the allelic level suggests that allele-specific regulation might exist alongside the individual-dependent regulation of expression discussed in the next paragraph. Indeed, concerning HLA-C alleles, Aguiar et al [28] showed that the rs 41561715-T SNP was associated to the HLA-C*04 lineage in lymphoblastoid cells. In our study, this SNP has a p-value of 3.1175e-06 and 0.0382403 for HLA-C in stimulated and unstimulated PBMCs, respectively. Unfortunately, we cannot comment further on lineage specific effects, as we do not have great enough sample size.

In all three cell cohorts used in this study (i.e., freshly isolated PBMCs, PBMCs kept overnight in culture medium with or without TNFα and IFNβ), we observed a coordinated HLA class I expression at the individual level rather than an allele-specific regulation. Indeed, calculation of allele-specific expression (ASE), measured as the proportion of the lowest expressed allele per locus per individual, revealed a very balanced and conserved expression of both alleles in heterozygotes with ratio close to 0.5 in uncultured cells as well as in cells cultured overnight with or without pro-inflammatory cytokines. The suggested individual regulated expression is sustained by high correlation and a very low variance of expression between paired alleles of the same PBMC sample. A random assignment of allelic pairs by permutations (even when constraining the permutations to keep the same HLA typing within each individual) showed that the observed ASE is significantly closer to the maximum of 0.5 (i.e., representing a fully balanced expression of both alleles) than any of the permutation replicates. The coordinated expression of alleles from distinct HLA class I loci in a given individual was best seen in Fig 3 when alleles belonging to the same haplotype were compared. This confirms our previous observation suggesting an association between HLA-C expression and extended HLA haplotypes [6]. The low number of samples that share the same haplotype(s) did not allow analyzing whether haplotype-specific regulating non-coding elements (eQTL’s) exists. Moreover, independently from haplotype segregation, eQTL analysis did not reveal variants affecting the bulk expression of the three HLA class I genes together. Each locus seemed thus to have his own regulatory transcription elements. The coordinated expression at the individual level observed in this study is also in agreement with previous studies of Vandidonck et al [33] and Lam et al. [45], who reported HLA haplotype-specific regulation of gene expression in distinct blastoid cell lines. Gene clustering and *cis* regulatory domains were proposed to explain the allelic co-expression. [45,46]. However, no higher co-expression of alleles within the same haplotypes compared to alleles on different haplotypes were reported by Aguiar et al [28]. Nevertheless, when they considered allele-specific expression (ASE), as we do in Figs 2 and 3, they observed a rather balanced expression profile for HLA-A, B and C alleles. This sustains our results suggesting the paired expression of HLA class I alleles in most individuals.

We are aware that analysis on different cohorts, namely T cells from the Blueprint consortium and PBMC’s represent different cell type composition and might not be directly comparable. Indeed, eQTLs were shown to be possibly related to differences in cell type composition across individuals [47]. Nevertheless, the eQTL association patterns we observed in T cells are similar to the eQTL association patterns in stimulated PMBCs for HLA-B and HLA-C genes. In addition, as seen in Fig 7, two SNPs (6:31274027 and 6: 31243785) were best predictor of HLA C expression. Overall, stimulated cells show stronger effect sizes when compared to unstimulated cells, suggesting that the effect of eQTLs on expression would be magnified in an active immune system.

In conclusion, our data showed that non-coding variations regulating HLA class I genes could be independent of the coding variations that define alleles. As the eQTLs identified in PBMCs mostly have low effect sizes, they may only imperfectly capture the true signals. Thus, confirmatory data on a larger cohort are warranted. More importantly, however, our results demonstrated a coordinated and paired expression of both alleles of the same locus in each individual, which is maintained under conditions of inflammation. Although our study was not designed to answer to this question, the poor correlation between eQTL and distinct HLA allelic expression could suggest that allelic regulation is mediated by sequences, which have not been preferentially selected during evolution. Only a larger sample size may allow the assignment of specific non-coding variants, which would be directly responsible for the intra-allelic variation in expression.

## Methodology

### Ethic statement

The studies involving human participants were reviewed and approved by Ethical committee of University Hospitals of Geneva (CER 06–208 and 08–208R). Written informed consent for participation was not required for this study in accordance with the national legislation and the institutional requirements.

### Cells

Peripheral blood mononuclear cells (PBMCs) were purified using standard Ficoll procedure from blood collected from healthy donors who were genotyped at loci HLA-A, B, C, DRB1, DRB3/4/5, DQB1 and DPB1 at high resolution by the Swiss National Reference Laboratory for Histocompatibility (LNRH), while searching for potential unrelated HSC donors.

To upregulate HLA expression, PBMCs were incubated in RPMI 1640 culture medium (Gibco) supplemented with 10mM L-glutamine 100 units/ml penicillin /streptomycin (Gibco) and 10% human AB serum (own preparation) overnight with 50 ng/ml TNFα and 100 ng/ml IFNβ (PrepoTech, London, UK) prior to RNA extraction.

### Immunofluorescence

HLA cell surface expression was determined on CD3^+^ T cells using the monoclonal antibodies APC-labeled anti-human CD3 (clone BW264/56) and FITC-labeled anti-HLA-ABC (clone REA230) (Milteny Biotec) and their corresponding isotype controls. Data acquisition was performed on gated mononuclear cells, using the ACCURI-C6 cytometer (BD) and the CFLOWPLUS analysis software (BD Bioscience, Allschwil, Switzerland).

### DNA extraction and high-resolution HLA typing

DNA was extracted on an automatic system (QIAGEN GmbH, Hilden, Germany) from Ficoll purified peripheral blood mononuclear cells (PBMCs). HLA typing was performed by reverse PCR-sequence-specific oligonucleotide microbeads arrays and high throughput sequencing (One Lambda, Canoga Park, USA).

### RNA extraction

Total RNA was extracted using the RNeasy Micro kit (Qiagen, Valencia, CA, USA) according to manufacturer’s instructions.

### Genotyping and imputation

The genotyping of the samples was conducted with the Illumina Infinium Global Screening Array v2.0. The genotype calls were done using Illumina GenomeStudio 2.0. We filtered variants with minor allele frequency (MAF) less than 1%, genotype missingness greater than 2.5%, and Hardy-Weinberg equilibrium (HWE) p-value less than 1e-5. This resulted in 484886 variants which were imputed using the Michigan Imputation Server with the HRC (Version r1.1 2016) reference panel. Post-imputation filters, r^2^ less than 0.3, MAF less than 1%, and HWE p-value less than 1e-6 were applied, resulting in 7924221 variants. Principle component analysis of the samples, together with 1000 genomes samples [48] revealed that 2 samples have non-European ancestry, thus were excluded from further analyses. The human reference genome build used was GRCh37.

### RNA-sequencing and quality control

RNA-sequencing was conducted on Illumina HiSeq 4000, according to manufacturer’s instructions. The RNA-seq QC was conducted according to Lappalainen T [49]. We also assessed the matching between the genotypes and the RNA-seq experiments using QTL tools mbv [50]. We observed low concordance at heterozygous sites (< 80%) for 6 samples, which were excluded from downstream analyses.

### HLA- allele-specific mRNA expression

The RNA sequencing method has previously extensively been discussed by Aguiar et al [28]. Briefly we used HLApers to create a personalized genome based on HLA alleles, mapped the RNA-seq reads against these genomes using STAR [51], and then quantified gene expression using Salmon [52] including allele specific HLA transcripts. The human reference genome build used was GRCh37, and gene annotation used was GENCODE v33 lifted over to b37GENCODE reference annotation for the human and mouse genomes [53]. We excluded genes that were not quantified in more than half of the samples.

### eQTL analyses

All analyses were conducted with QTLtools [46]. We calculated population principal components (PCs) from genotypes and technical RNA-seq covariate PCs, using QTLtools pca, and all PCs were centered and scaled. The number of RNA-seq was chosen such that they maximized the number of cis eQTLs genome-wide. We used 5 RNA-seq PCs and 3 population PCs, as technical covariates in PBMCs, and 30 RNA-seq PCs and 3 population PCs in T cells. Cis eQTL analysis was conducted using QTLtools cis with 1000 permutations.

### Graphs

Graphs were generated using R version 4.0.2.

### Statistics

Statistical paired *t* tests were performed with R version 4.0.2 and GraphPad prism version 8.0.

## Supporting information

S1 Fig Gradient expression of HLA antigens. HLA class I RNA expression measured in samples of PBMCs obtained from 63 individuals is plotted as tpm (transcript per million). Alleles are grouped according to their serological specificity (i.e., HLA-A, B and C antigens, as indicated on the horizontal axis). Each dot represents the expression of a given allele/antigen in one individual.
(TIF)

S2 Fig Correlation of expression between pairs of HLA alleles. The RNA expression of pairs of HLA -A, B and C alleles measured in 63 different PBMC samples are plotted as transcript per million (tpm) against each other. The Spearman coefficient ϱ and associated p-value are indicated.
(TIF)

S3 Fig TNFα/IFNβ induced HLA class I upregulation per allele. HLA class I RNA expression measured in 56 different PBMC samples stimulated with or without TNFα+IFNβ overnight is plotted for each HLA-A, B and C allele taken individually. The numbers in the plots represent median fold upregulation ratios. Alleles are given on the top of each plot.
(TIF)

S4 Fig Cytofluorometric gating strategy of TNFα/IFNβ induced HLA class I upregulation. HLA cell surface expression on gated CD3^+^ lymphocytes (upper panels) from PBMC stimulated over night without (grey histograms) or with TNFα/IFNβ (light blue histograms). HLA class I typing of the corresponding PBMC’s are: HLA-A*03:01,32:01 HLA*B 08:01,44:03 HLA*C 04:01,07:01 (left lower panel), HLA-A*03:01,24:02 HLA*B 07:02,38:01 HLA*C 07:02,12:03 (middle lower panel), HLA-A*02:01,24:02 HLA*B 39:06,44:02 HLA*C 05:01,07:02 (right lower panel). Corresponding mean fluorescence intensities (MFI) are indicated.
(TIF)

S5 Fig Correlation of expression between pairs of HLA alleles. The RNA expression of pairs of HLA -A, B and C alleles measured in 56 different PBMC samples stimulated with or without TNFα+IFNβ overnight are plotted against each other. The Spearman coefficient ϱ is indicated.
(TIF)

S6 Fig The correlation between the non-coding eQTL variants and the coding variants defining alleles. The correlation (r^2^, calculated from 1000 genomes European samples) between the best PBMC variants (indicated on the top of the panels) corresponding to a T-cell independent eQTL signal and all the coding variants responsible for an individual’s allele type of HLA-A, HLA-B, and HLA-C.
(TIF)
