# Supplementary figures and images for: Regulation of HLA class I expression by non-coding gene variations
Source: PLoS Genet. 2022 Jun 6;18(6):e1010212. doi: 10.1371/journal.pgen.1010212 (PMC9170083; doi:10.1371/journal.pgen.1010212)

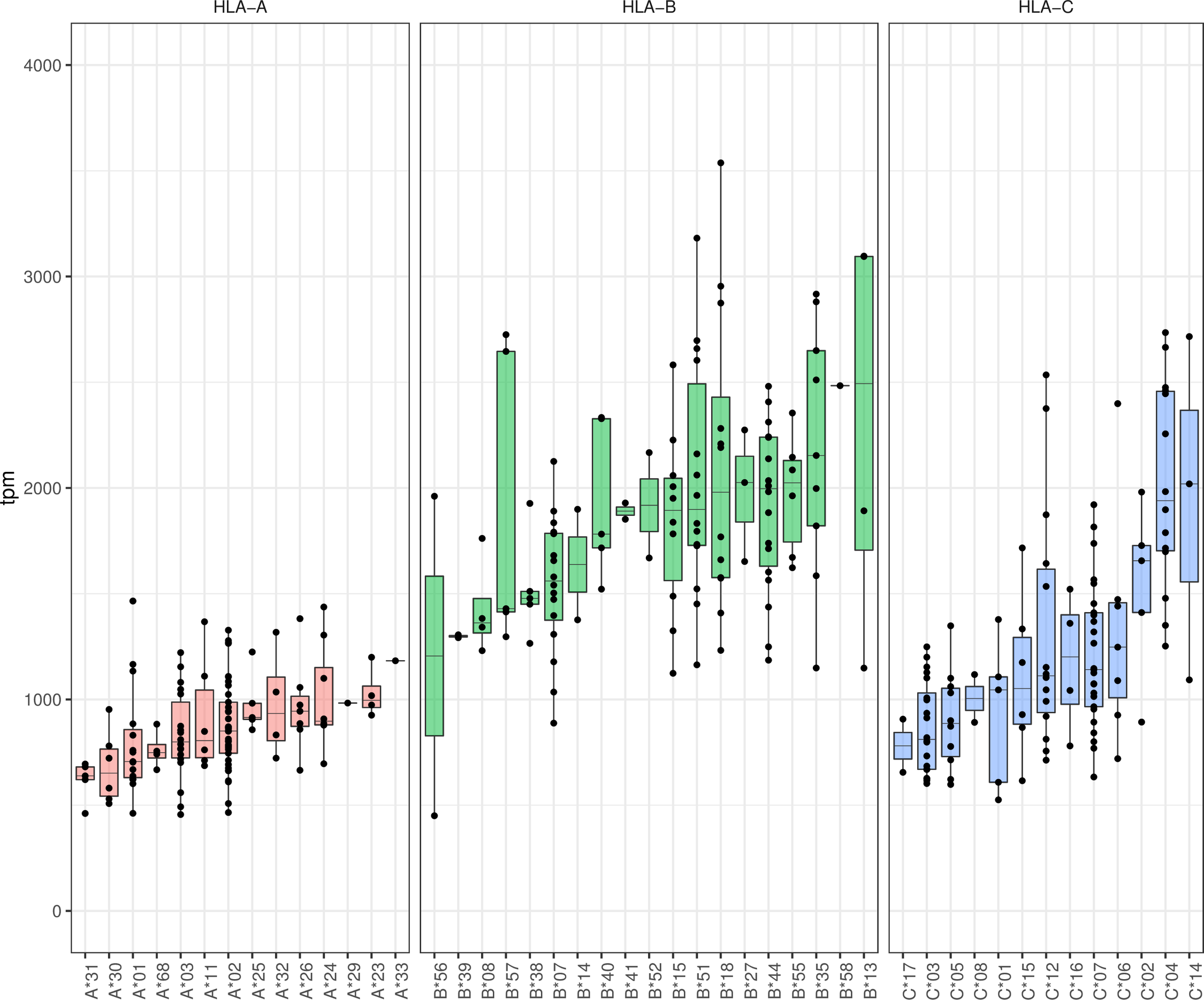

Supplement: S1 Fig — HLA class I RNA expression measured in samples of PBMCs obtained from 63 individuals is plotted as tpm (transcript per million). Alleles are grouped according to their serological specificity (i.e., HLA-A, B and C antigens, as indicated on the horizontal axis). Each dot represents the expression of a given allele/antigen in one individual. (TIF) [file pgen.1010212.s001.tif]

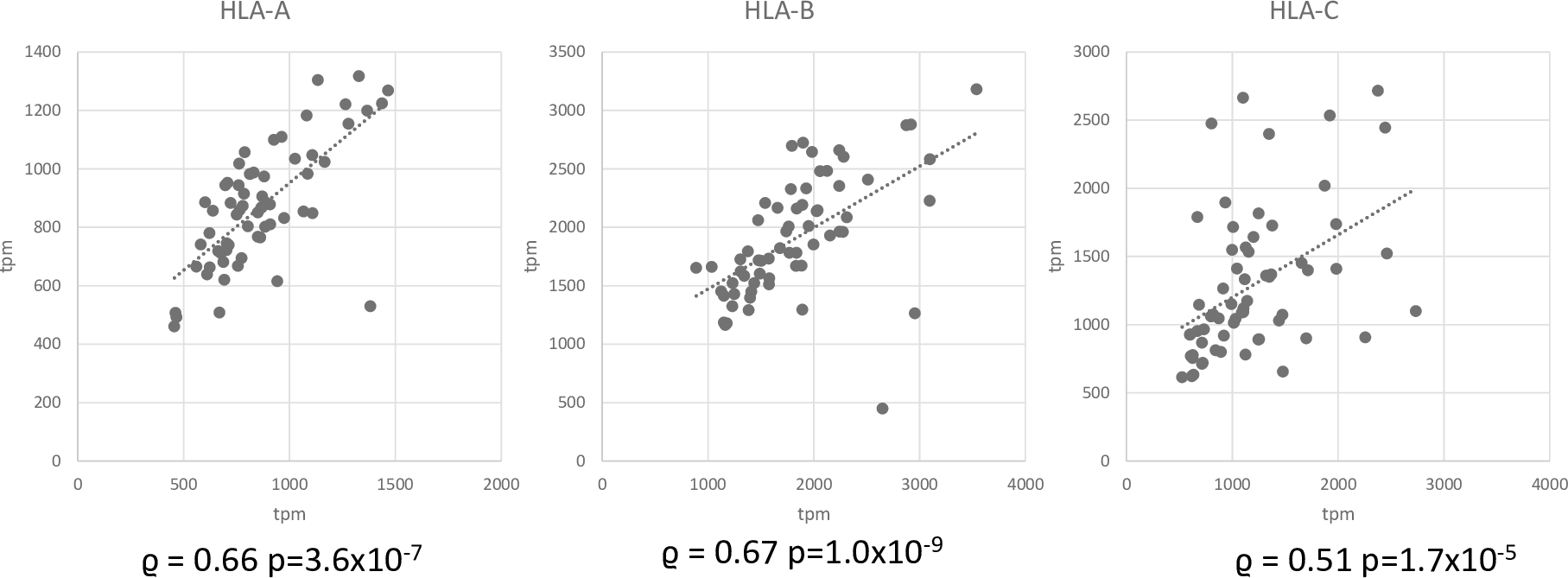

Supplement: S2 Fig — The RNA expression of pairs of HLA -A, B and C alleles measured in 63 different PBMC samples are plotted as transcript per million (tpm) against each other. The Spearman coefficient ϱ and associated p-value are indicated. (TIF) [file pgen.1010212.s002.tif]

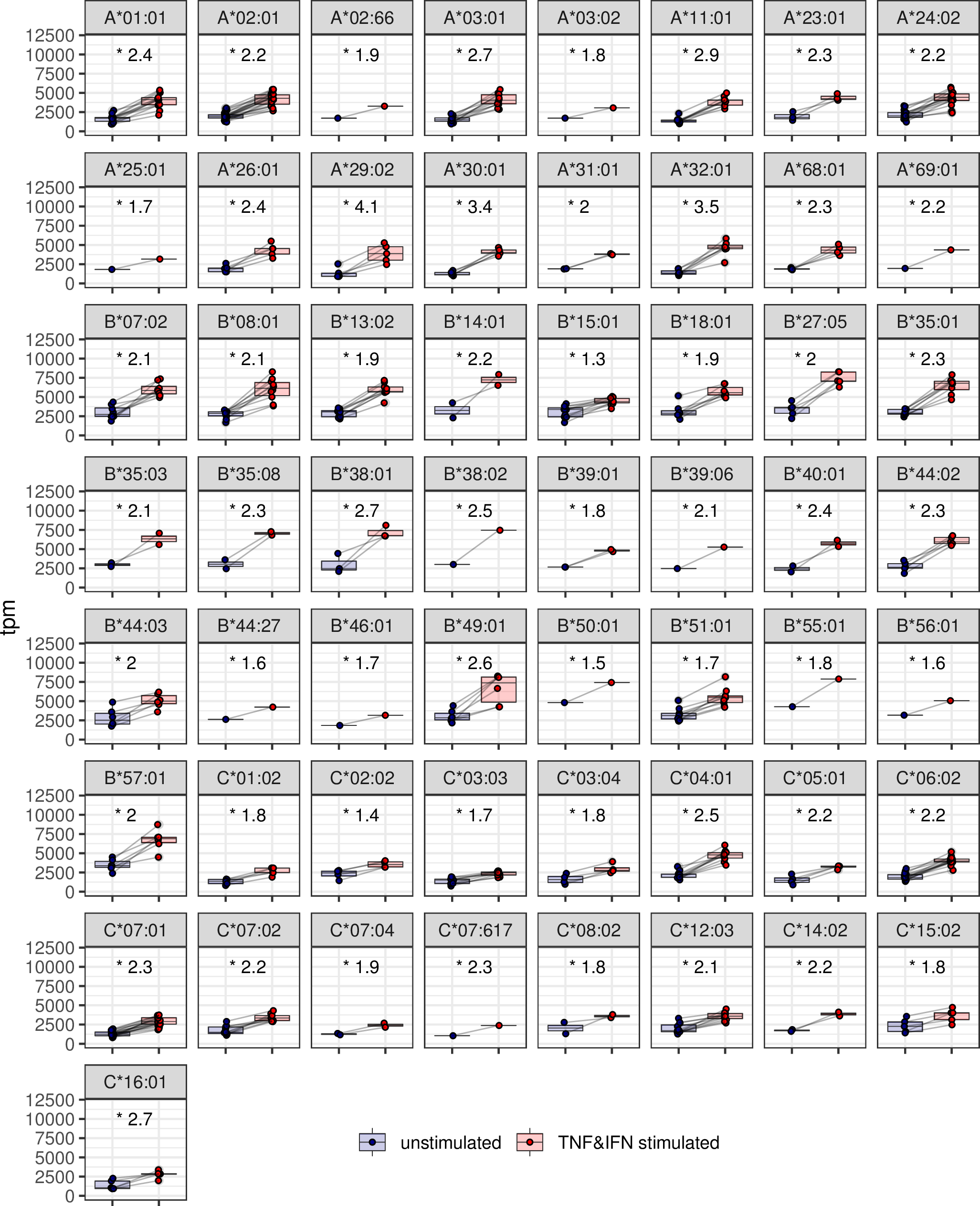

Supplement: S3 Fig — HLA class I RNA expression measured in 56 different PBMC samples stimulated with or without TNFα+IFNβ overnight is plotted for each HLA-A, B and C allele taken individually. The numbers in the plots represent median fold upregulation ratios. Alleles are given on the top of each plot. (TIF) [file pgen.1010212.s003.tif]

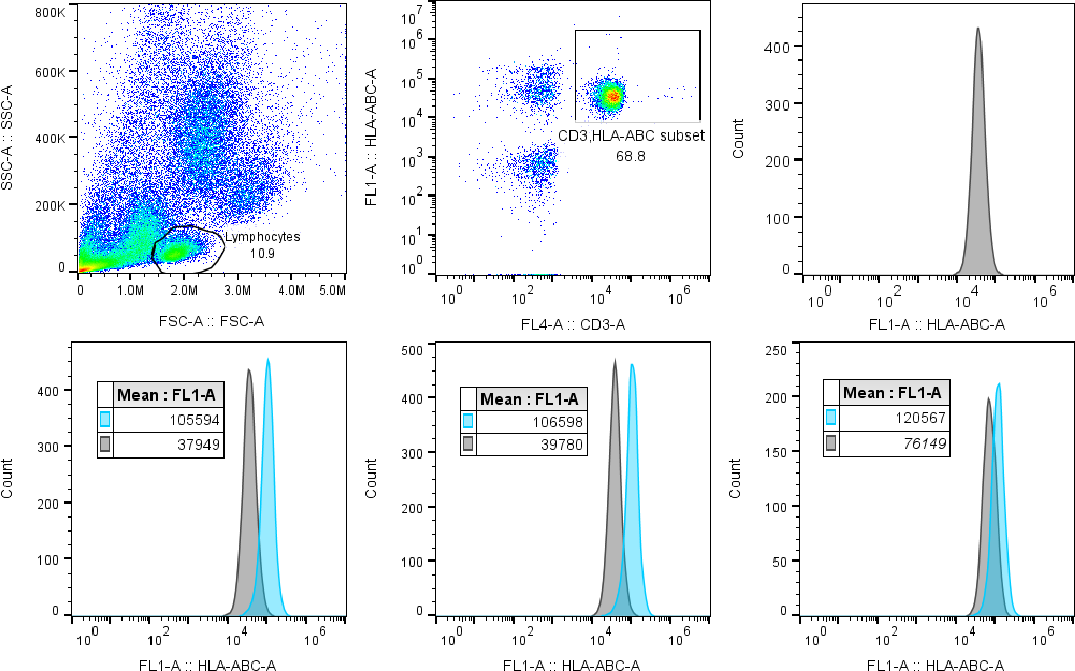

Supplement: S4 Fig — HLA cell surface expression on gated CD3+ lymphocytes (upper panels) from PBMC stimulated over night without (grey histograms) or with TNFα/IFNβ (light blue histograms). HLA class I typing of the corresponding PBMC’s are: HLA-A*03:01,32:01 HLA*B 08:01,44:03 HLA*C 04:01,07:01 (left lower panel), HLA-A*03:01,24:02 HLA*B 07:02,38:01 HLA*C 07:02,12:03 (middle lower panel), HLA-A*02:01,24:02 HLA*B 39:06,44:02 HLA*C 05:01,07:02 (right lower panel). Corresponding mean fluorescence intensities (MFI) are indicated. (TIF) [file pgen.1010212.s004.tif]

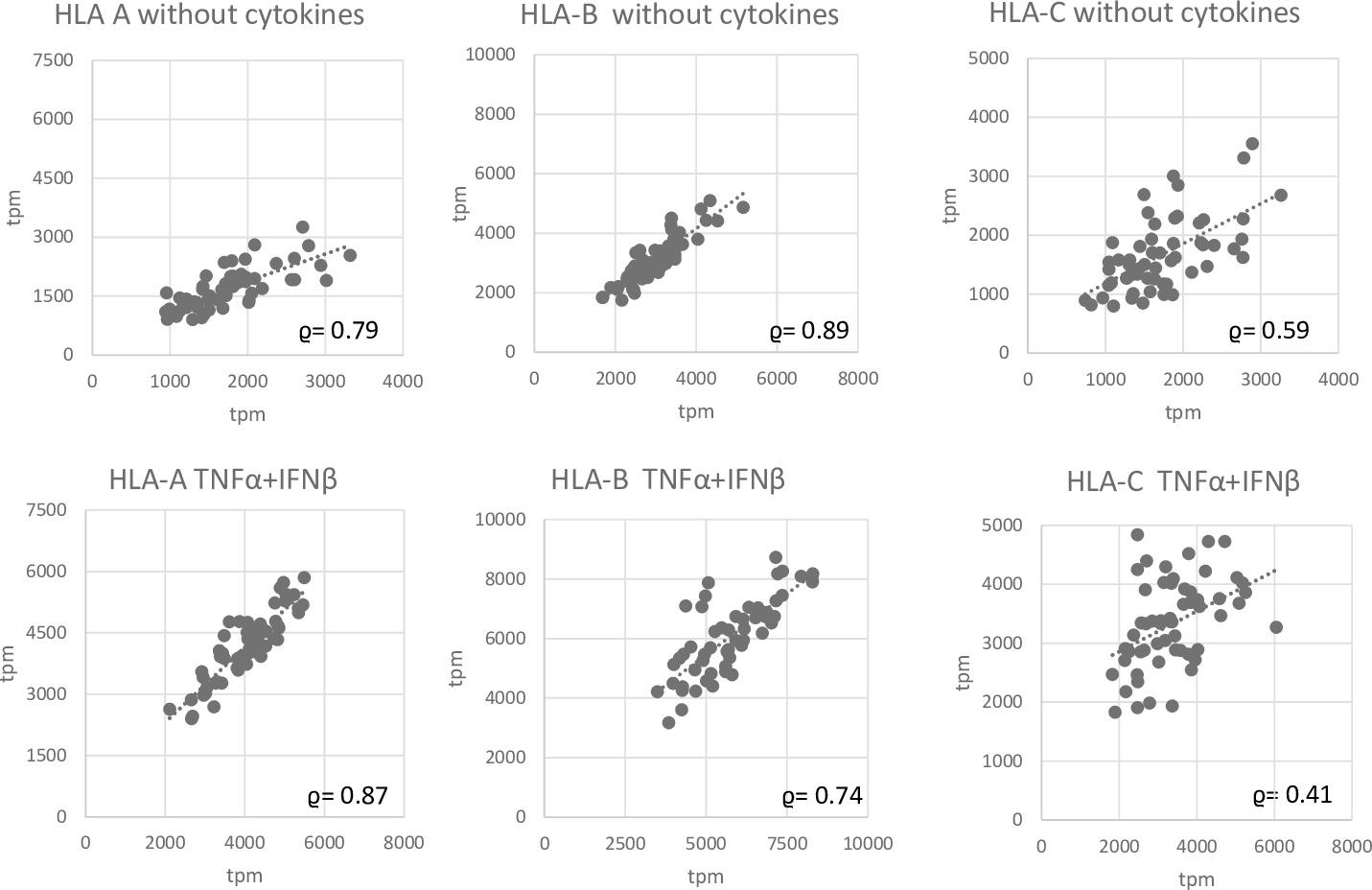

Supplement: S5 Fig — The RNA expression of pairs of HLA -A, B and C alleles measured in 56 different PBMC samples stimulated with or without TNFα+IFNβ overnight are plotted against each other. The Spearman coefficient ϱ is indicated. (TIF) [file pgen.1010212.s005.tif]

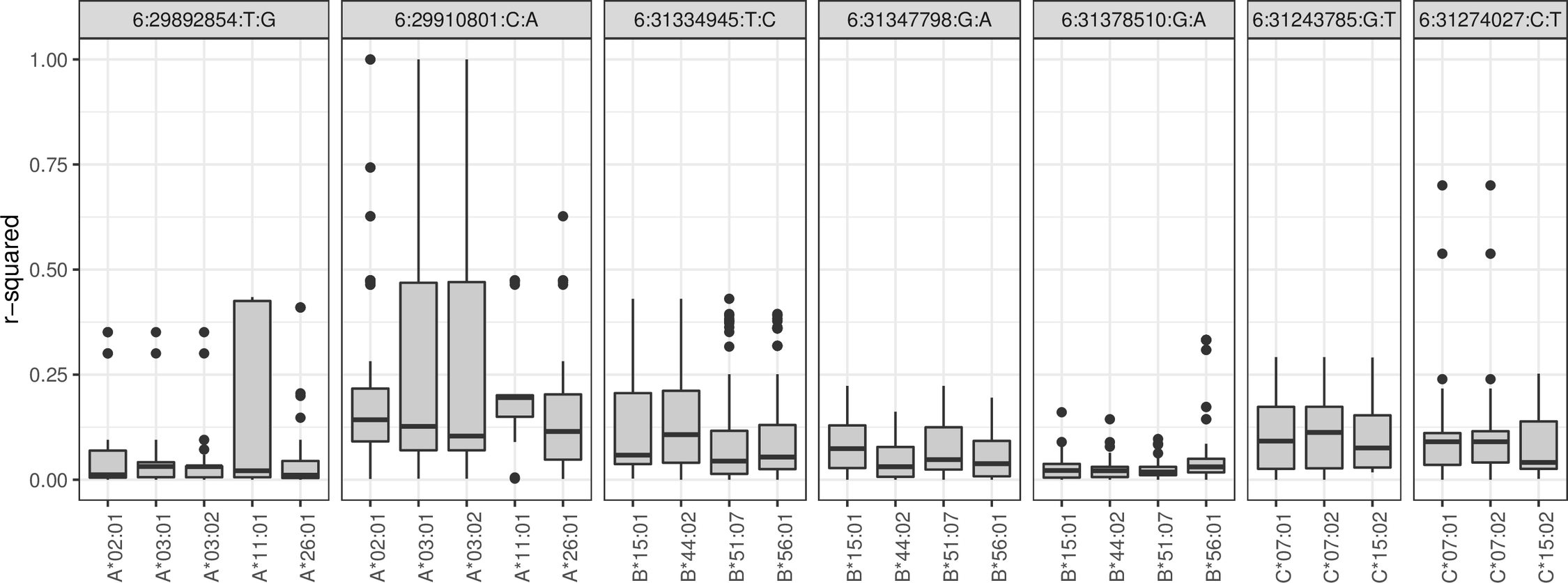

Supplement: S6 Fig — The correlation (r2, calculated from 1000 genomes European samples) between the best PBMC variants (indicated on the top of the panels) corresponding to a T-cell independent eQTL signal and all the coding variants responsible for an individual’s allele type of HLA-A, HLA-B, and HLA-C. (TIF) [file pgen.1010212.s006.tif]
